# Chronic kidney disease under non-dialysis dependent, hemodialysis, peritoneal dialysis and kidney transplant treatment: Body composition data

**DOI:** 10.1016/j.dib.2020.106601

**Published:** 2020-11-28

**Authors:** Natália Tomborelli Bellafronte, Luisa Maria Diani, Lorena Vega-Piris, Paula Garcia Chiarello, Guillermina Barril Cuadrado

**Affiliations:** a Post-graduate Program in Health Sciences, Ribeirão Preto Faculty of Medicine, University of São Paulo, Bandeirantes Avenue, 3900, Ribeirão Preto, 14049-190 São Paulo, Brazil; b Nutrition and Metabolism Undergraduate Course, Ribeirão Preto Faculty of Medicine, University of São Paulo, Bandeirantes Avenue, 3900, Ribeirão Preto, São Paulo 14049-190, Brazil; c Methodology Unit, Instituto de Investigación Sanitaria del Hospital Universitario de la Princesa, Diego de León Street, 62, Madrid 28006, Spain; d Department of Health Sciences, Ribeirão Preto Faculty of Medicine, University of São Paulo, Bandeirantes Avenue, 3900, Ribeirão Preto, São Paulo 14049-190, Brazil; e Nephrology Department, Hospital Universitario de la Princesa, Diego de León Street, 62, Madrid 28006, Spain

**Keywords:** Bioelectrical impedance, Body composition, Chronic kidney disease, Dual energy X-ray absorptiometry, Fat free mass, Fat mass

## Abstract

This article presents a dataset of body composition in chronic kidney disease (CKD) non-dialysis-dependent (NDD), hemodialysis (HD) and peritoneal dialysis (PD) (for at least 3 months), and kidney transplantation (KTx) (for at least 6 months) patients. The data were collected as part of a PhD research project, an observational cross-sectional study followed by a prospective analysis (about 6 months later). Adult CKD patients (18≤age≤60 years old) from a tertiary hospital were recruited: CKD in stages 3b to 5 for NDD patients; PD patients without peritonitis in the last 30 days; HD patients in 4-hour dialysis session, 3 times per week, through an arteriovenous fistula; and KTx patients with CKD in stages 1 to 3a. Patients with presence of amputated limbs or an electronic implant, wheelchair user or inpatient, body weight above 140 kg or BMI higher than 40 kg/m^2^, acute infections, cancer diagnosis, acquired immunodeficiency syndrome, and others that could alter body composition were excluded. The dataset in this publication consist of some clinical measurements for characterization of the sample, body composition measurements by dual-energy X-ray absorptiometry and by bioelectrical impedance spectroscopy in tetra-polar whole-body wrist to ankle (BISWB) and segmental (BISSEG) protocols of 266 CKD patients, being 137 men and 129 women; 81 in NDD treatment, 83 in HD, 24 in PD, and 80 in KTx. Measurements were performed consecutively by the same professional after an 8-hour fast, empty urinary bladder, drainage of the peritoneal dialysate, and just after the midweek hemodialysis session. To analyze differences among subgroups according to sex and CKD treatment, unpaired T test or ANOVA and Chi-square, adjusted by Bonferroni post-test, were applied. Agreement in fat free mass and fat mass measurements between BISWB and BISSEG, for cross-sectional data and for body composition changes (prospective measurement – cross-sectional measurement), was checked using intraclass correlation coefficient and 95% confidence intervals. Agreement on individual level was evaluated using the Bland-Altman method with limits of agreement. The data can be valuable in the study of body composition in CKD under all types of treatment and also for agreement analysis among body composition measurements by different instruments and techniques. The data are analysed and interpreted in the research article Bellafronte et al., 2020 [1].

## Specifications Table

**Table:** 

Subject	Nutrition and Nephrology
Specific subject area	Body composition in chronic kidney disease
Type of data	Table Figure
How data were acquired	Body composition were assessed applying a fan-beamed dual energy x-ray absorptiometry (DXA) using the model Hologic (Hologic Discovery WI®, USA) and also by bioelectrical impedance spectroscopy applying the model Body Composition Monitor (BCM®, Fresenius Medical Care, DEU) performed in a tetra-polar unilateral whole-body wrist-to-ankle (BISWB) and segmental (BISSEG) protocols.
Data format	Raw Analyzed Filtered
Parameters for data collection	Chronic kidney disease (CKD) patients (18≤age≤60 years old) from a tertiary hospital were recruited: CKD in stages 3b to 5 for non-dialysis dependent group; peritoneal dialysis patients without peritonitis in the last 30 days and hemodialysis patients (4-hour dialysis session, 3 times per week, through an arteriovenous fistula), both in dialysis for at least 3 months; and kidney transplant patients for at least 6 months with CKD in stages 1 to 3a.
Description of data collection	Clinical and biochemical data and self-declared ethnicity were collected from electronic medical records up to 10 days prior to the study. Anthropometric (weight at the nearest 0.1 kg, height and waist circumference at the nearest 0.1 cm), and BIS and DXA measurements were performed consecutively in the same day by the same technician after an 8-hour fast, empty urinary bladder, drainage of the peritoneal dialysate, and just after the midweek hemodialysis session. Measurements were performed with patients wearing a light gown and without shoes.
Data source location	Institution: University of São Paulo, Ribeirão Preto Faculty of Medicine City/Town/Region: Ribeirão Preto, São Paulo State Country: Brazil
Data accessibility	Tables and figure are available with this article. The dataset is available as a supplementary material (excel data basis Bellafronte NT data set). Will be also available in a public repository through the following data repository once the article is accepted for publication. Repository name: Mendeley Data Data identification number: Reserved DOI: 10.17632/cv3dxvxxv8.1 Direct URL to data: the URLs/accession numbers/DOIs will be available only after acceptance of the manuscript for publication so that we can ensure their inclusion before publication
Related research article	Authors’ names: Natalia Tomborelli Bellafronte MD, Luisa Maria Diani, Lorena Veja-Piris MD, Paula Garcia Chiarello PhD, Guillermina Barril Cuadrado PhD. Title: Comparison between dual-energy x-ray absorptiometry and bioelectrical impedance for body composition measurements in adults with chronic kidney disease: a cross-sectional, longitudinal and multi-treatment analysis. Journal: Nutrition. In Press. https://doi.org/10.1016/j.nut.2020.111059[1].

## Value of the Data

• This dataset consist of measurements of body composition by different equipments and techniques in CKD patients under NDD, HD, PD and KTx treatment. Analysing these data may offer valuable insight on the association of body composition with clinical conditions in CKD, including type of treatment.
• Medical, dieticians and others health professionals who are studying the altered body composition of CKD patients and also the ones who are studying how to assess body composition by different techniques and equipments may benefit from the data.
• New statistical models can be developed to understand the relationships between CKD and body composition changes. Also, differences between measurements of body composition by different protocols or equipments could be better understood.

## 1 Data Description

The dataset consists of measurements from 266 subjects. The raw data could be found in “Bellafronte NT data set” document, an excel sheet. In “Plan 1” are all raw data with the description of any data and codes applied in the first line of each column. The first column lists non-identifiable Subject ID; the others columns have data about: age, ethnicity, presence of Diabetes Mellitus, presence of Systemic Arterial Hypertension, presence of Dislipidemia, dialysis or kidney transplant time, estimated glomerular filtration rate, KT/V, creatinine, weight, body mass index and waist circumference. Data from bioelectrical impedance by whole-body wrist-to-ankle (BISWB) measurements are: resistance, reactance, phase angle, body cell mass, extracellular water, intracellular water, total body water, extra to intracellular water ratio, overhydration, fat free mass, fat free mass index, fat mass and fat mass index. Data from bioelectrical impedance by segmental (BISSEG) analysis are: fat free mass, fat free mass index, fat mass and fat mass index. Data by dual energy X-ray absorptiomnetry are: appendicular lean mass index, lean mass, lean mass index, fat free mass, fat free mass index, fat mass and fat mass index.

Table 1 presents the descriptive analysis of total sample stratified by sex and CKD subgroup, with analysis of ANOVA (quantitative variables) and Chi-Square (qualitative variables) between the same sex stratified by CKD subgroups with adjusted p by Bonferroni post-test; and analysis of unpaired student T-test (quantitative variables) and Chi-Square (qualitative variables) between male and female sex in total sample and in each CKD subgroup.

**Table 1:** Descriptive analysis of total sample stratified by sex and CKD subgroup.

		NDD				HD				PD				KTx				TotalMale sample		Total female sample	
		Menn = 46		Womenn = 37		Menn = 35		Womenn = 44		Menn = 8		Womenn = 15		Menn = 48		Womenn = 33		n = 137		n =129	
Variables	Categories	Χ	SD	Χ	SD	Χ	SD	Χ	SD	Χ	SD	Χ	SD	Χ	SD	Χ	SD	Χ	SD	Χ	SD
**Age (years)**	**-**	49^ab^	10	48^a^	10	44^b^	12	49^a^	8	37^c^	12	42^b^	12	50^a^	8	48^a^	9	47	11	48	9
**Ethnicity % (n)**	**White**	76 (35)^a^		70 (26)^a^		66 (23)^a^		75 (33)^a^		50 (4)^a^		87 (13)^a^		71 (34)^a^		79 (26)^a^		70 (96)		76 (98)	
**DM % (n)**	**Present**	33 (15)^a^		27 (10)^ab^		9 (3)^b^		16 (7)^a^		0		7 (1)^a^		42 (20)^a^		45 (15)^b^		28 (38)		26 (33)	
**SAH % (n)**	**Present**	74 (34)^a^		73 (27)^a^		63 (22)^a^		70 (31)^a^		62 (5)^a^		60 (9)^a^		77 (37)*^a^		45 (15)*^a^		71 (98)		64 (82)	
**DLP % (n)**	**Present**	26 (12)^a^		19 (7)^a^		11 (4)^a^		18 (8)^a^		12 (1)^a^		7 (1)^a^		31 (15)^a^		21 (7)^a^		23 (32)		18 (23)	
**Dialysis or kidney transplant time (month)**	**-**	NA		NA		65^a^	55	81^a^	67	11^b^	9	18^b^	20	96	64	86	57	NA		NA	
**eGFR (ml/min/1.73m^2^)**	**-**	19.29^a^	9.27	17.80^a^	7.53	NA		NA		NA		NA		71.25^b^	16.78	69.05^b^	20.79	NA		NA	
**KT/V**	**-**	NA		NA		1.48*^a^	0.19	1.84*^a^	0.72	2.72^b^	0.52	2.53^b^	0.51	NA		NA		NA		NA	
**Cr (mg/dl)**	**-**	4.07*^a^	1.31	3.39*^a^	1.21	12.71*^b^	2.46	9.36*^b^	2.89	8.29^c^	2.86	8.77^c^	3.28	1.54^d^	12.14	1.01^d^	0.23	5.56	4.89	5.45	4.14
**Weight (kg)**	**-**	84*^a^	16	69*^a^	16	69^b^	14	64^ab^	13	79*^abc^	15	59*^b^	9	76*^c^	12	64*^ab^	11	77*	15	65*	13
**BMI (kg/m^2^)**	**-**	28^a^	5	28^a^	7	24^b^	4	26^ab^	5	26^ab^	4	24^b^	3	27^a^	4	26^a^	4	27	5	26	5
**WC (cm)**	**-**	105^a^	13	100^a^	15	94^b^	13	97^ab^	12	98^ab^	12	91^b^	9	102*^a^	10	97*^a^	8	100*	13	97*	12

BMI, body mass index; CKD, chronic kidney disease; Cr, creatinine; DLP, Dyslipidemia; DM, diabetes Mellitus; eGFR, estimated glomerular filtration rate; HD, hemodialysis; KTx, kidney transplant; NA, not applicable; NDD, non-dialysis dependent; PD, peritoneal dialysis; SAH, Systemic arterial hypertension; WC, waist circumference.Ethnicity: white or non-white (black, mulatto or dark skinned). ^abc^: ANOVA (quantitative variables) and Chi-Square (qualitative variables) between the same sex stratified by CKD subgroups with adjusted p by Bonferroni test, p≤0.013 (values without a common letter differ significantly).

⁎ unpaired Student t-test (quantitative variables) and Chi-Square (qualitative variables) between male and female sex in total sample and in each CKD subgroup, p≤0.05.

Table 2 presents the bioelectrical impedance data by BISWB of total sample stratified by sex and CKD subgroup, with analysis of ANOVA (quantitative variables) and Chi-Square (qualitative variables) between the same sex stratified by CKD subgroups with adjusted p by Bonferroni post-test; and analysis of unpaired student T-test (quantitative variables) and Chi-Square (qualitative variables) between male and female sex in total sample and in each CKD subgroup.

**Table 2:** Bioelectrical impedance data by BISWB of total sample stratified by sex and CKD subgroup.

		NDD				HD				PD				KTx				Total male sample		Total female sample	
		Menn = 46		Womenn = 37		Menn = 35		Womenn = 44		Menn = 8		Women n = 15		Menn = 48		Womenn = 33		n = 137		n =129	
Variables	Categories	Χ	SD	Χ	SD	Χ	SD	Χ	SD	Χ	SD	Χ	SD	Χ	SD	Χ	SD	Χ	SD	Χ	SD
**Re 50 kHz (ohm)**	**-**	436*^a^	66	549*^a^	115	538*^b^	87	630*^b^	77	476*^abc^	66	580*^a^	±90	476*^c^	62	593*^a^	73	479*	80	592*	95
**Xc 50 kHz (ohm)**	**-**	48*^a^	12	55*^a^	15	61^b^	±15	60^a^	14	51^ac^	8	57^a^	15	52*^c^	8	57*^a^	9	53*	12	58*	13
**PhA (°)**	**-**	6.19*^a^	1.06	5.64*^a^	0.77	6.34*^a^	±0.94	5.46*^a^	1.19	6.17^a^	0.65	5.60^a^	1.01	6.30*^a^	0.78	5.50*^a^	0.68	6.26*	0.91	5.54*	0.93
**PhA classification^1^ % (n)**	**LowPhA**	43 (20)*^a^		19 (7)*^a^		37 (13)^a^		32 (14)^a^		37 (3)^a^		33 (5)^a^		37 (18)^a^		30 (10)^a^		40 (54)*		28 (36)*	
**BCM (kg)**	**-**	28.68*^a^	5.21	18.59*^a^	3.17	25.22*^b^	±4.50	15.64*^bc^	4.69	28.62*^ab^	5.37	18.78*^a^	3.22	26.48*^ab^	5.28	15.83*^c^	2.78	27.02*	5.21	16.90*	3.92
**ECW (L)**	**-**	19.42*^a^	2.97	14.57*^a^	2.84	15.53*^b^	±3.26	12.49*^b^	2.21	18.67*^ac^	2.86	13.23*^ab^	2.00	17.32*^c^	2.21	12.93*^b^	1.84	17.65*	3.16	13.28*	2.43
**ICW (L)**	**-**	23.46*^a^	3.20	17.04*^a^	2.33	20.63*^b^	±2.79	15.17*^b^	3.18	23.28*^ac^	3.47	16.26*^ab^	2.25	21.71*^bc^	3.05	15.13*^b^	1.81	22.11*	3.24	15.82*	2.65
**TBW (L)**	**-**	42.89*^a^	5.46	31.61*^a^	4.93	36.17*^b^	±5.55	27.68*^b^	5.13	42.01*^ac^	6.08	29.50*^ab^	3.99	39.03*^c^	4.96	28.06*^b^	3.47	39.77*	5.92	29.12*	4.82
**ECW/ICW**	**-**	0.83^a^	0.11	0.85^a^	0.10	0.75*^b^	±0.11	0.83*^a^	0.11	0.80^a^	0.07	0.81^a^	0.08	0.80*^ab^	0.07	0.85*^a^	0.07	0.80*	0.10	0.84*	0.10
**OH (L)**	**-**	1.05*^ac^	2.01	0.09*^a^	1.38	-0.18^b^	±1.99	-0.66^b^	1.49	1.30^a^	0.70	0.25^a^	1.51	0.39*^bc^	1.02	-0.14*^ab^	0.87	0.51*	1.72	-0.20*	1.35
**Dehydration** **% (n)**	**OH< -1.1L**	6 (3)^a^		19 (7)^a^		34 (12)^b^		43 (19)^b^		0		13 (2)^a^		2 (1)^a^		6 (2)^a^		12 (15)*		23 (30)*	
**Hiperhydration % (n)**	**OH> +1.1L**	37 (17)^a^		22 (8)^ab^		20 (7)^ab^		14 (6)^ab^		37 (3)^a^		27 (4)^a^		12 (6)^b^		6 (2)^b^		24 (33)		15 (20)	

BCM, body cell mass; BISWB, spectroscopy multifrequency bioelectrical impedance by whole-body analysis; CKD, chronic kidney disease; ECW, extra cellular water; ICW, intracellular water; OH,overhydration; PhA, phase angle; Re, resistance; TBW, total body water; Xc, reactance. Bioelectrical impedance data by BISWB. ^1^: PhA<6.11° for men and <5.14° for women^(25)^. ^abc^: ANOVA (quantitative variables) and Chi-Square (qualitative variables) between the same sex stratified by CKD subgroups with adjusted p by Bonferroni test, p≤0.013 (values without a common letter differ significantly).

⁎ unpaired Student t-test (quantitative variables) and Chi-Square (qualitative variables) between male and female sex in total sample and in each CKD subgroup, p≤0.05.

Table 3 presents body composition analysis of total sample stratified by sex and CKD subgroup, with data by DXA and BISWB, applying analysis of ANOVA (quantitative variables) and Chi-Square (qualitative variables) between the same sex stratified by CKD subgroups with adjusted p by Bonferroni post-test; and analysis of unpaired Student t-test (quantitative variables) and Chi-Square (qualitative variables) between male and female sex in total sample and in each CKD subgroup.

**Table 3:** Body composition analysis of total sample stratified by sex and CKD subgroup.

	NDD				HD				PD				KTx				Total male sample		Total female sample	
	Menn = 46		Womenn = 37		Menn = 35		Womenn = 44		Menn = 8		Womenn = 15		Menn = 48		Womenn = 33		n = 137		n =129	
Variables	Χ	SD	Χ	SD	Χ	SD	Χ	SD	Χ	SD	Χ	SD	Χ	SD	Χ	SD	Χ	SD	Χ	SD
**DXA**																				
**ALMI (kg/m^2^)**	8.08*^a^	1.18	6.37*^a^	1.28	7.13*^b^	0.98	5.66*^b^	0.77	7.78*^abc^	1.49	5.67*^ab^	0.88	7.59*^c^	0.98	5.73*^b^	0.81	7.65*	1.13	5.88*	1.00
**LM (kg)**	50.88*^a^	8.03	36.50*^a^	7.27	43.95*^b^	7.71	33.02*^b^	5.89	48.13*^ab^	9.18	31.74*^b^	4.34	45.74*^b^	6.49	32.04*^b^	5.05	47.15*	7.95	33.62*	6.21
**LMI (kg/m^2^)**	17.53*^a^	2.54	14.57*^a^	2.84	15.53*^b^	2.19	13.38*^b^	1.45	15.66*^ab^	2.54	12.72*^b^	1.47	16.28*^b^	2.02	13.32*^b^	1.88	16.47*	2.40	13.63*	2.12
**FFM (kg)**	52.87*^a^	8.31	37.90*^a^	7.47	45.59*^b^	7.95	34.23*^b^	6.13	50.26*^ab^	9.55	32.95*^b^	4.50	47.53*^b^	6.68	33.21*^b^	5.22	48.99*	8.23	34.87*	6.42
**FFMI (kg/m^2^)**	18.21*^a^	2.61	15.09*^a^	2.92	16.08*^b^	2.37	13.86*^b^	1.49	16.33*^ab^	2.61	13.19*^b^	1.49	16.90*^b^	2.06	13.78*^b^	1.92	17.09*	2.46	14.11*	2.18
**FM (kg)**	24.48^a^	8.62	26.15^a^	9.34	17.27*^b^	8.72	24.25*^ab^	8.50	22.07^ab^	7.92	20.85^b^	5.63	22.01*^a^	6.67	25.26*^a^	7.30	21.63*	8.36	24.65*	8.25
**FMI (kg/m^2^)**	8.45*^a^	2.99	10.48*^a^	3.81	6.11*^b^	3.08	9.94*^ab^	3.63	7.16^ab^	2.54	8.41^b^	2.36	7.85*^a^	2.50	10.47*^a^	2.88	7.57*	2.94	10.05*	3.41
**BISWB**																				
**FFM (kg)**	49.15*^a^	7.47	33.53*^a^	4.61	44.03*^b^	6.90	29.25*^b^	7.31	49.81*^ac^	7.84	33.72*^a^	4.87	45.71*^bc^	7.71	29.29*^b^	3.94	46.68*	7.67	31.01*	5.91
**FFMI (kg/m^2^)**	16.95*^a^	2.49	13.35*^a^	1.75	15.55*^b^	2.01	11.79*^b^	2.25	16.27*^ab^	2.47	13.39*^a^	1.64	16.25*^ab^	2.44	12.19*^b^	1.74	16.31*	2.29	12.54*	2.03
**FM (kg)**	24.54^a^	11.08	26.17^a^	11.38	22.52*^b^	9.93	25.72*^a^	8.62	20.58^ab^	9.11	18.34^b^	5.49	21.84^b^	9.09	25.17^a^	7.58	21.82*	10.22	24.85*	9.21
**FMI (kg/m^2^)**	8.44*^a^	3.75	10.45*^a^	4.60	6.53*^b^	3.48	10.56*^a^	3.70	6.60^ab^	2.88	7.39^b^	2.29	7.79*^b^	3.36	10.41*^a^	2.97	7.62*	3.55	10.12*	3.78

ALMI, appendicular lean mass index; BISWB,spectroscopy multifrequency bioelectrical impedance by whole-body analysis; CKD, chronic kidney disease; DXA, dual-energy X-ray absorptiometry; FFM, fat free mass, FFMI, fat free mass index; FM, fat mass; FMI, fat mass index; HD, hemodialysis; KTx, kidney transplant; LM, lean mass; LMI, lean mass index.; NDD, non-dialysis dependent; PD, peritoneal dialysis. ^abc^: ANOVA (quantitative variables) and Chi-Square (qualitative variables) between the same sex stratified by CKD subgroups with adjusted p by Bonferroni test, p≤0.013 (values without a common letter differ significantly).

⁎ unpaired Student t-test (quantitative variables) and Chi-Square (qualitative variables) between male and female sex in total sample and in each CKD subgroup, p≤0.05.

Table 4 presents agreement between BISWB and BISSEG in total sample stratified by sex, about cross sectional and prospective data, applying analysis of intraclass correlation coefficient between BISWB and BISSEG measurements with 95% Confidence interval. Limits of agreement by Bland-Altman analysis was also applied. Bias was calculates as BISWB-BISSEG measurements.

**Table 4:** Agreement between BISWB and BISSEG in total sample stratified by sex.

	Men									Women								
					Bland-Altman analysis			ICC analysis						Bland-Altman analysis			ICC analysis	
	BISWB		BISSEG		Bias^1^					BISWB		BISSEG		Bias^1^				

Variables	Χ	SD	Χ	SD	Χ	SD	LOA Lower;upper	ICC value	95%CI Lower;upper	Χ	SD	Χ	SD	Χ	SD	LOA Lower;upper	ICC value	95%CI Lower;upper
**Cross-sectional data n = 137**										**Cross-sectional data n = 129**								
**FFM (kg)**	46.6	7.67	61.59	4.59	-14.83	6.13	11.06; 18.37	0.142	-0.062; 0.416	31.01	5.91	42.57	3.19	-11.50	4.80	-20.91;-2.09	0.122	-0.060; 0.373
**FFMI (kg/m^2^)**	16.31	2.39	21.59	1.67	-5.25	2.26	-9.67; -0.82	0.096	-0.058; 0.309	12.54	2.03	17.31	1.47	-4.75	2.20	-9.07; -0.44	0.050	-0.045; 0.175
**FM (kg)**	21.82	10.22	13.70	4.16	8.37	8.15	-7.60; 24.34	0.288	-0.048; 540	24.85	9.21	24.92	4.04	0.00	7.47	-14.64; 14.64	0.448	0.297; 0.576
**FMI (kg/m^2^)**	7.62	3.55	4.85	1.68	2.85	2.77	-2.58; 8.29	0.330	-0.043; 0.588	10.12	3.78	10.21	2.16	-0.06	3.03	-6.00; 5.88	0.517	0.377; 0.633
**∆ data n = 47**										**∆ data n = 40**								
**FFM (kg)**	-0.40	4.09	-1.31	2.65	1.03	3.50	-5.83; 7.89	0.473	0.152; 0.705	-0.67	3.02	-0.19	2.48	-0.52	2.59	-5.60; 4.56	0.518	0.232; 0.723
**FFMI (kg/m^2^)**	-0.12	1.46	-0.37	1.16	0.19	1.31	-2.37; 2.76	0.424	0.095; 0.668	-0.29	1.22	-0.15	0.97	-0.15	1.03	-2.17; 1.86	0.500	0.184; 0.723
**FM (kg)**	0.36	4.44	0.53	2.88	-0.11	3.42	-6.82; 6.60	0.581	0.303; 0.767	-0.44	3.37	-0.39	2.01	-0.09	2.74	-5.47; 5.28	0.495	0.183; 0.716
**FMI (kg/m^2^)**	0.23	1.48	0.23	1.03	-0.05	1.20	-2.40; 2.30	0.590	0.319; 0.719	-0.26	1.38	-0.08	0.86	-0.21	1.17	-2.51; 2.09	0.488	0.183; 0.707

BISWB, spectroscopy multifrequency bioelectrical impedance by whole-body analysis; BISSEG, spectroscopy multifrequency bioelectrical impedance by segmental analysis; FFM, fat free mass; FFMI, fat free mass index; FM, fat mass; FMI, fat mass index; ICC, intraclass correlation coefficient between BISWB and BISSEG measurements with 95% Confidence interval (95%CI); LOA, limits of agreement by Bland-Altman analysis (lower limit; upper limit).

1 : Bias calculates as BISWB-BISSEG measurements. ∆ data: body composition changes evaluated as prospective – cross-sectional measurements.

**Fig. 1:**
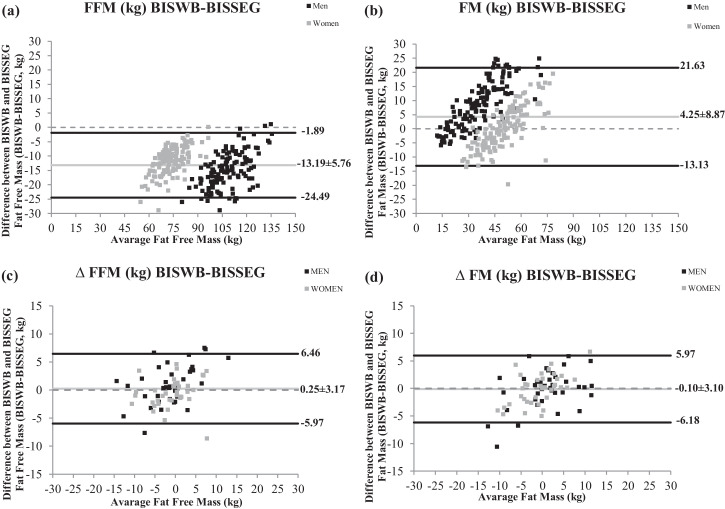
Bland–Altman plot with sex stratification for comparison between BISWB and BISSEG. (a) Fat free mass (FFM) data by cross-sectional measurements, (b) Fat mass (FM) data by cross-sectional measurements, (c) FFM data by prospective – cross-sectional measurements (∆), (d) FM data by prospective – cross-sectional measurements (∆). Solid black horizontal lines are limits of agreement; solid grey horizontal line is the median and stand deviation of bias between methods.

Fig. 1 presents Bland–Altman plot with sex stratification for comparison between BISWB and BISSEG, for cross-sectional and prospective data of fat free mass, fat free mass index, fat mass and fat mass index.

## 2 Experimental Design, Materials and Methods

CKD patients (18≤age≤60 years old) from a tertiary hospital were recruited: CKD in stages 3b to 5 for NDD group; PD patients without peritonitis in the last 30 days and HD patients (4-hour dialysis session, 3 times per week, through an arteriovenous fistula), both in dialysis for at least 3 months; and KTx patients for at least 6 months with CKD in stages 1 to 3a.

Clinical and biochemical data and self-declared ethnicity were collected from electronic medical records up to 10 days prior to the study. Anthropometric (weight at the nearest 0.1 kg, height and waist circumference at the nearest 0.1 cm), and BIS and DXA measurements were performed consecutively in the same day by the same technician after an 8-hour fast, empty urinary bladder, drainage of the peritoneal dialysate, and just after the midweek hemodialysis session. Measurements were performed with patients wearing a light gown and without shoes.

Body composition were assessed using fan-beamed DXA applying the model Hologic (Hologic Discovery WI®, USA) [2] and also by bioelectrical impedance spectroscopy applying the model Body Composition Monitor (BCM®, Fresenius Medical Care, DEU) performed in a tetra-polar unilateral whole-body wrist-to-ankle [3] and segmental [4] protocols.

To analyze differences among subgroups according to sex and CKD treatment, unpaired T-test or ANOVA (quantitative variables) and Chi-square (qualitative variables), adjusted by Bonferroni post-test were applied.

Agreement in FFM and FM _measurements_ between BISWB and BISSEG, for cross-sectional data and for body composition changes (prospective measurement – cross-sectional measurement) was checked using intraclass correlation coefficient and 95% confidence intervals [5]. Agreement on individual level was evaluated using the Bland-Altman method with limits of agreement.

Significance was set at p ≤ 0.05 except if adjustment for multiple comparisons were necessary.

All statistics were done using IBM SPSS Statistics 23 (IBM, NY, USA).

## Ethics Statement

The local Ethics Committee approved the study (protocol number: 2053045). All participants read and signed the informed consent form before the procedures began.

## Declaration of Competing Interest

The authors declare that they have no known competing financial interests or personal relationships which have, or could be perceived to have, influenced the work reported in this article.

## Appendix Supplementary materials

Image, application 1
